# Stepwise dissection of *Plasmodium falciparum* merozoite invasion of the human erythrocyte

**DOI:** 10.1186/1475-2875-9-S2-O25

**Published:** 2010-10-20

**Authors:** David Riglar, Dave Richard, Michelle Boyle, Danny Wilson, Fiona Angrisano, Lynne Turnbull, Cynthia Whitchurch, Alan Cowman, James Beeson, Stuart Ralph, Jake Baum

**Affiliations:** 1 Walter and Eliza Hall Institute of Medical Research, Parkville Victoria 3052, Melbourne, Australia; 2 iThree Institute, University of Technology Sydney, Ultimo NSW 2007, Australia; 3 Bio21 Molecular Sciences and Biotechnology Institute, University of Melbourne, Parkville Victoria 3010, Melbourne, Australia

## Background

A critical step in establishing malaria parasite infection is the ability of the blood stage merozoite to invade erythrocytes. However, much of our understanding about the cell biology of this process has remained unchanged since seminal work, more than 30 years ago, defined the steps of entry by light and electron microscopy. These studies were, however, only possible using merozoites from simian and avian malaria parasite species, given the poor viability of merozoites from human parasites, specifically *Plasmodium falciparum.* In contrast, critical invasion proteins have been best described for human or mouse parasite species. Thus our understanding about the molecular and cellular coordination of the entire process of invasion is still largely unknown. Towards addressing this gap, we recently developed a method for harvesting viable *P. falciparum* merozoites, permitting detailed investigation of the molecular events of merozoite invasion [1].

## Results

Using super resolution and immunoelectron microscopy of *P. falciparum* merozoites we place the core invasion machinery in context as the parasite enters the red blood cell. We establish that irreversible merozoite attachment to the erythrocyte surface is the trigger for all subsequent invasion events, which include: secretion of apical rhoptries, surface-protein shedding and actomyosin motor activation. Irreversible attachment is therefore the master switch for merozoite entry, with the tight junction acting as a nexus around which invasion is orchestrated.

## Conclusions

Detailed imaging analysis allows us to propose a complete model for merozoite invasion and permits understanding of not only the mechanics of invasion but also the mechanism of action of drug or other strategies that perturb the process. The ability to understand this key step in malaria disease establishment provides a general platform to evaluate strategies that target merozoite entry for malaria therapy.

## References

[B1] Boyle MJ Wilson DW Richards JS Riglar DT Tetteh KK Conway DJ Ralph SA Baum J Beeson JG Isolation of viable Plasmodium falciparum merozoites to define erythrocyte invasion events and advance vaccine and drug development Proc Natl Acad Sci U S A 2010 107 14378 8 10.1073/pnas.1009198107 20660744 PMC2922570

